# Phenotypic Aging and Sexual Dimorphism in C57BL/6J, SAMR1, and SAMP8 Mice: A Comparative Study

**DOI:** 10.1155/mi/1198691

**Published:** 2026-07-21

**Authors:** Luiz Adriano Damasceno Queiroz, Renata Spalutto Fontes, Mariana de Araujo Oliveira, Kamilla Costa Pantoja, Josiane Betim Assis, Ywa Perpetuo Socorro Toda Tavares, Rafael dos Santos Barros, Walter Miguel Turato, Anderson Sá-Nunes, Naima Moustaid-Moussa, Stephen Fernandes Rodrigues, Joilson O. Martins

**Affiliations:** ^1^ Laboratory of Immunoendocrinology, Department of Clinical and Toxicological Analyses, School of Pharmaceutical Sciences, São Paulo University (FCF/USP), São Paulo, Brazil, usp.br; ^2^ Biomodels Research Center of the Faculty of School of Pharmaceutical Sciences and Chemistry Institute, São Paulo University (FCF-IQ/USP), São Paulo, Brazil, usp.br; ^3^ Laboratory of Experimental Immunology, Department of Immunology, Institute of Biomedical Sciences, São Paulo University (ICB/USP), São Paulo, Brazil, usp.br; ^4^ Multi-User Preclinical Imaging Center, Department of Clinical and Toxicological Analyses, School of Pharmaceutical Sciences, São Paulo University (FCF/USP), São Paulo, Brazil, usp.br; ^5^ Nutrigenomics, Inflammation and One Health Research Laboratory, School of Veterinary Medicine, Texas Tech University, Amarillo, Texas, USA, ttu.edu; ^6^ Institute for One Health Innovation, Texas Tech University and Texas Tech Health Sciences Center, Lubbock, Texas, USA, ttu.edu; ^7^ Laboratory of Vascular Nanopharmacology, Department of Pharmacology, Institute of Biomedical Sciences of University-Sao Paulo (ICB/USP), Sao Paulo, Brazil, usp.br

**Keywords:** aging, C57BL/6J, SAMP8, SAMR1

## Abstract

Aging research increasingly relies on animal models to study the multifactorial processes of senescence. This study aimed to compare the physiological and metabolic parameters of C57BL/6J mice, a reference strain for biomedical research, with two strains commonly used to assess senescence: senescence‐accelerated resistant 1 (SAMR1) and senescence‐accelerated prone 8 (SAMP8) mice, over a 1‐year period, in order to enhance our understanding of their utility in aging research. We focused on reproductive performance, glucose metabolism, bone density, hematological profiles, and overall longevity. SAMR1 mice exhibited higher prolificacy compared to C57BL/6J and SAMP8, with no significant differences in other reproductive parameters. Both SAMR1 males and females were larger and heavier, while C57BL/6J mice consumed less food relative to their body weight. Bone density decreased in all male groups from the fourth to the tenth month, while SAMP8 females showed an unexpected increase. C57BL/6J mice presented higher glucose levels, with SAMP8 males showing similar glucose intolerance and insulin resistance. Hematological analyses revealed sexual dimorphism, with SAMP8 males showing a distinct immunosenescence profile. Longevity varied by strain and sex, with SAMP8 males and SAMR1 females exhibiting shorter lifespans. These findings suggest that SAMR1 animals, despite sharing a genetic background with SAMP8, display a phenotype more akin to normal aging, supporting their use as a control in aging studies. Moreover, SAMP8’s accelerated senescence makes it a valuable model for immunosenescence research. The study highlights the importance of considering strain‐ and sex‐specific differences when working on aging research.

## 1. Introduction

The global increase in the elderly population has become a major issue, with the World Health Organization estimating that nearly 2 billion people over the age of 65 will be in 2050. This demographic shift poses significant challenges for pension and healthcare systems, particularly in countries with larger aging populations, due to the rising costs of age‐related diseases [1, 2]. In this context, understanding the physiological and biochemical changes associated with the aging process is crucial for developing strategies to mitigate and control age‐related diseases. Animal models are therefore essential tools for investigating the mechanisms underlying aging.

Laboratory mice are widely used in aging research due to their practical and biological advantages, including ease of handling and breeding, short lifespan, substantial genomic homology to humans, and low maintenance costs. These attributes facilitate their use across diverse experimental settings, and decades of domestication have led to the development of numerous inbred and outbred strains tailored to specific scientific questions [3–5].

Aging models are broadly classified as natural (chronological) or accelerated. A commonly used natural‐aging strain is C57BL/6J, which typically lives two to 3 years under standard laboratory conditions [1]. In contrast, senescence‐accelerated mouse strains (SAMP series) are characterized by the early onset of age‐related phenotypes; for example, senescence‐accelerated prone 8 (SAMP8) mice exhibit a reduced median lifespan of approximately 1 year. SAMP strains carry distinct genetic alterations (including variants in the *Abcb1a* gene, which encodes P‐glycoprotein, an ATP‐dependent efflux transporter) that contribute to their accelerated senescence phenotype [6]. Although SAMP8 and related strains are widely used, several alternative models of accelerated or induced senescence exist, such as D‐galactose treatment, *Rps9* D95N mutation, progeria models, mitochondrial DNA polymerase mutants, total‐body irradiation, ozone exposure, and chronic jet lag [1].

Among standard laboratory strains, C57BL/6J is one of the most extensively characterized and widely used models. However, each inbred strain displays distinct phenotypic traits, and an increasing number of studies now employ genetically heterogeneous or outbred mice (e.g., four‐way crosses, F1 hybrids) as “wild‐type” counterparts [7]. In contrast, SAMP8 and its counterpart senescence‐accelerated resistant 1 (SAMR1) are less broadly characterized outside the senescence‐accelerated mouse research community. Both lines originate from the inbreeding of AKR/J progenitors, yielding nine senescence‐prone (P1–P9) and four senescence‐resistant (R1–R4) strains with distinct pathophysiological profiles. SAMP strains display accelerated senescence, while SAMR strains show features more consistent with normal aging; SAMR1 is commonly used as a control for SAMP8 studies, as both strains originate from the same progenitors and therefore share a closely related genetic background, differing primarily in the mutations associated with the accelerated senescence phenotype [8–10].

Together, C57BL/6J, SAMR1, and SAMP8 represent different manifestations of aging, a multifactorial process shaped by genetic and environmental inputs that ultimately influence lifespan [11]. Natural aging is a gradual process marked by the progressive decline of physiological resistance and homeostatic capacity, leading to increased disease susceptibility and eventual death. In contrast, accelerated aging results from genetic mutations or experimental insults that precipitate a rapid loss of adaptive capacity and premature, often multi‐organ, functional decline [1, 12]. Consequently, accelerated models do not fully recapitulate natural aging, and these phenotypic disparities must be taken into account when interpreting the experimental outcomes.

Accordingly, when using SAMP8 as a model of accelerated senescence, appropriate controls are essential. SAMR1 is commonly used because it shares a genetic background with SAMP8; however, parallel comparison with a widely used wild‐type strain that undergoes natural aging (e.g., C57BL/6J) is recommended to better assess how closely SAMR1 reflects physiological aging and to compare the biological performance across models.

The characterization of senescence was performed through a multidimensional approach, employing the biogerontological parameters applied in the SAM model studies by Takeda [8]. In addition to analyzing the survival curve and reproductive performance, aging progression was quantified using biomarkers of physiological and functional health, considering the variations inherent in sexual dimorphism. In this context, our study aims to provide an updated phenotypic framework for these models, serving as a practical reference for the scientific community. This effort is particularly relevant given the growing importance of geriatric research in a rapidly aging global population, where extending not only lifespan but also healthspan remains a central goal supported by advances in aging research.

## 2. Material and Methods

### 2.1. Ethical Statement and Animal Housing

This study was conducted in strict accordance with the principles and guidelines established by the National Council for the Control of Animal Experimentation (CONCEA, Brazil). The experimental protocol was reviewed and approved by the Institutional Animal Care and Use Committee (IACUC) of the School of Pharmaceutical Sciences at the University of São Paulo, Brazil (Protocol Number CEUA/FCF/646).

### 2.2. Animal Models and Environmental Conditions

Male and female C57BL/6J, SAMR1, and SAMP8 mice were utilized. Animals were housed in individually ventilated cages maintained at a controlled temperature of 23 ± 2°C and a relative humidity of 55 ± 10%. A 12‐h light/dark cycle was regulated by a digital timer. Cages were provided with autoclaved aspen wood shavings for bedding and environmental enrichment, consisting of cardboard rolls and nesting material. Because the mean lifespan of SAMP8 is 1 year, the experimental timeline was established around this period to allow direct, time‑matched comparisons among the three strains; selected physiological and behavioral assessments were therefore conducted at months 4 and 10 to capture changes occurring at an earlier and a later stage of the SAMP8 lifespan relative to the naturally aging C57BL/6J and SAMR1 strains.

### 2.3. Dietary Specifications and Composition

Mice were granted ad libitum access to filtered, autoclaved water and an irradiated commercial rodent diet (Nuvilab CR1, QUIMTIA SA, Curitiba, Brazil). The nutritional composition of the Nuvilab CR1 diet per kilogram consisted of crude protein (min. 200 g), ether extract (min. 50 g), crude fiber (max. 50 g), mineral matter (max. 90 g), moisture (max. 125 g), calcium (10–12 g), and phosphorus (min. 7000 mg). The amino acid profile included lysine (min. 11 g) and methionine (min. 4000 mg), with BHT (125 mg) added as an antioxidant.

The vitamin fortification per kilogram comprised vitamin A (min. 15,000 UI), vitamin D3 (min. 2300 UI), vitamin E (min. 60 UI), vitamin K3 (min. 2 mg), vitamin B1 (min. 15 mg), vitamin B2 (min. 10 mg), vitamin B6 (min. 8 mg), vitamin B12 (min. 60 mcg), niacin (min. 60 mg), calcium pantothenate (min. 17 mg), folic acid (min. 2 mg), biotin (min. 0.2 mg), choline (min. 1950 mg), and Vitamin C (min. 30 mg). The mineral content per kilogram included sodium (min. 2700 mg), iron (min. 50 mg), manganese (min. 60 mg), zinc (min. 70 mg), copper (min. 12 mg), iodine (min. 3 mg), selenium (min. 0.2 mg), cobalt (min. 0.8 mg), and fluorine (max. 70 mg).

### 2.4. Evaluation of Reproductive Performance

The following parameters were analyzed over a 6‐month period postmating using 10 breeding pairs per strain: I) Fertility rate: the number of females that gave birth divided by the total number of mated females, multiplied by 100. II) Time interval between births: the duration between consecutive births. III) Prolificacy: the average number of offspring born per litter. IV) Mortality rate: the number of offspring deaths before weaning per litter, expressed as a percentage. V) End age of reproductive phase: the final age at the end of the reproductive phase. VI) Number of litters per breeding pair: the total number of litters produced per breeding pair. Data were collected throughout six gestations within one generation.

### 2.5. Metabolic Parameters

For the characterization of SAMP8, SAMR1, and C57BL/6J mice, weekly and monthly observations of the following parameters were conducted [7]: I) Animal weight: measurements were recorded weekly from the first week of life until 12 weeks of age, with individual weights documented. After this period, weight measurements were taken once a month until the end of the experiment. II) Food consumption: assessed in the 13th week of life, based on the average amount of food (g) consumed per mouse over a period of 5 days. III) Blood glucose levels: measured monthly from the 13th week until the end of the experiment using the Accu‐Chek Active glucose monitor, with measurements consistently taken in the late afternoon.

### 2.6. Survival Curve

To evaluate animal longevity, inspections were carried out every 2 days to assess health problems. Animals were euthanized via cervical dislocation following light sedation with isoflurane‐saturated air if they exhibited severe debilitating signs that compromised their survival beyond an additional 48 h. Severe debilitation was defined by the presence of more than one of the following clinical signs: (1) inability to eat or drink; (2) severe lethargy, indicated by a lack of response, such as reluctance or inability to walk when gently stimulated to move; (3) lack of coordination or gait disturbance; (4) rapid weight loss over 1 week or more (more than 30% of body weight); or (5) a severely ulcerated or bleeding tumor. The age at which the debilitated animal was euthanized was recorded as the best estimate of its natural lifespan. Animals found dead in their cages were considered to have died from natural causes and were included in the survival calculations [13–15].

### 2.7. Bone Density Analysis

The animals were anesthetized with isoflurane at 2%–3% in medicinal oxygen and placed in a ventral decubitus position within the MSFX‐Pro system (Bruker BioSpin Corporation, Billerica, MA, USA), remaining under anesthesia for the entire duration of the process. Imaging was conducted according to the parameters listed in Table 1. Subsequent analysis of the obtained images was performed using Bruker Molecular Imaging Software. Four rectangular regions of interest (ROI) perpendicular to the left femur of each animal were defined. The bone density in each of these regions was calculated independently using the algorithm provided by the software [16].

**Table 1:** Parameters for image acquisition using Bruke molecular imaging software for bone density analysis.

Variable	Image
Electric current	35 KVP
Field of view	90 mm
Diaphragm aperture	2.5
Focal plane	12 mm
*x*‐Binning	0
*y*‐Binning	0
X‐ray filter	0.4 mm

### 2.8. Insulin Tolerance Test (ITT) and Glucose Tolerance Test (GTT)

ITT and GTT were performed after 6 h of fasting at the 4^th^ and 10^th^ months of life. Initial blood glucose levels were determined, followed by an intraperitoneal injection of human insulin (Humulin, Fegersheim, France) (0.75 UI/kg). Blood glucose levels were measured via tail vein blood at 15, 30, 60, 90, and 120 min after the injection [9]. In another set of experiments, initial blood glucose levels were determined, followed by an intraperitoneal injection of glucose solution (Thermo Fisher Scientific, Rockford, IL, USA) (1 g/kg). Blood glucose levels were measured via tail vein blood at 15, 30, 60, 90, and 120 min after the injection [13].

### 2.9. Hematological Analyses

Blood samples were collected via the tail vein at the 4^th^ and 10^th^ months of life. Samples of EDTA‐anticoagulated blood (1:10) were used to determine the following hematological parameters: red blood cells (RBC), hemoglobin (HGB), hematocrit (HCT), mean corpuscular volume (MCV), mean corpuscular hemoglobin (MCH), MCH concentration (MCHC), red cell distribution width (RDW), platelets (PLTs), leukocytes (WBC), lymphocytes (Lymphs), monocytes (Mons), and granulocytes (Grans); and the percentage of lymphocytes (Lymph%), monocytes (Mon%), and granulocytes (Gran%). All analyses were performed using an automated hematology counter (BC‐2800Vet Mindray, Shenzhen, GD, China) [13].

### 2.10. Statistical Analyses

Statistical analyses were performed using GraphPad 6 software (San Diego, CA, USA). Data are presented as mean ± standard error of the mean (SEM) using analysis of variance (ANOVA), two‐way for variables including age (4 M vs 10 M), and one‐way for the other evaluations, followed by Tukey’s test. The significance level was set at *p* ≤ 0.05.

## 3. Results

### 3.1. C57BL/6J, SAMR1, and SAMP8 Have No Differences in Fertility

**Figure 1:**
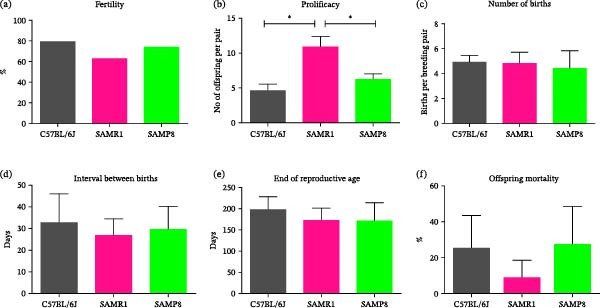
Reproductive efficiency parameters in C57BL/6J, SAMR1, and SAMP8 mice: (a) fertility rate (%), (b) prolificacy (number of offspring born per breeding pair), (c) number of births per breeding pair, (d) interval between births (days), (e) end of reproductive phase (days), and (f) offspring mortality rate (%). Results are expressed as the mean ± standard deviation.  ^∗^Represents the statistical difference between the groups evaluated by one‐way ANOVA Tukey’s test, considering *p* < 0.05 (6–10 animals per group).

To evaluate the reproductive efficiency of C57BL/6J, SAMR1, and SAMP8 mice, we analyzed several parameters, including fertility (Figure 1a), prolificacy (Figure 1b), the total number of litters per breeding pair over the reproductive life (Figure 1c), the interval between births (Figure 1d), the end of reproductive age (Figure 1e), and offspring mortality per breeding pair (Figure 1f). Among these parameters, the only statistically significant difference was observed in prolificacy, which was higher in SAMR1 compared to the other two strains (Figure 1b). No significant differences were found in the remaining reproductive parameters.

### 3.2. C57BL/6J, SAMR1, and SAMP8 Show Sexual Dimorphism in the Expression of Their Physiological Phenotype

**Figure 2:**
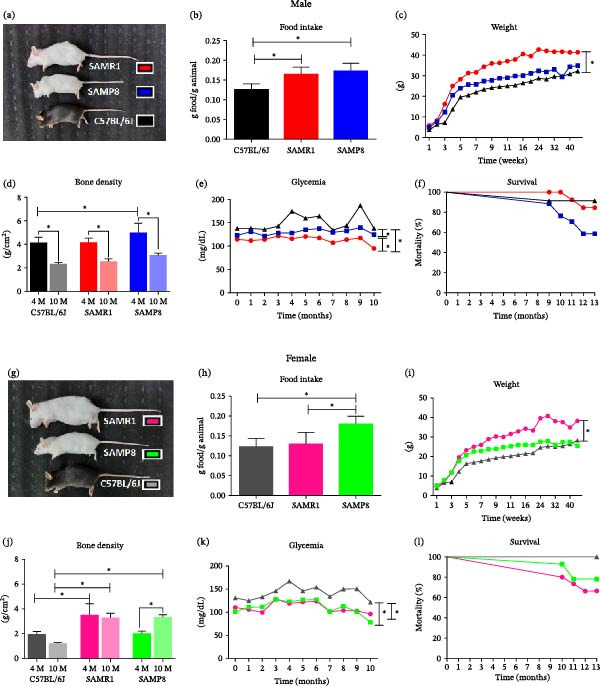
Characterization of physiological metrics in C57BL/6J, SAMR1, and SAMP8 mice. Panels show male data in (a–f) and female data in (g–l): (a, g) Representative photographs of each strain; (b, h) food intake per animal; (c, i) body weight progression; (d, j) bone mineral density at 4 and 10 months; (e, k) glycemia over time; and (f, l) survival analysis. Results are expressed as the mean ± standard deviation.  ^∗^Represents the statistical difference between the groups evaluated by two‐way ANOVA Tukey’s test for d and j, and one‐way for all others, considering *p* < 0.05 (4–5 animals per group for bone density experiments, and 9–12 animals per group for the others).

After assessing the reproductive profile of the strains, we proceeded with the phenotypic characterization of the animals over a period of 13 months. We observed that both male and female SAMR1 mice were visibly larger (Figure 2a,g) and heavier (Figure 2c,i) than the other strains. Relative food intake was lower in C57BL/6J males compared to other strains; however, among females, the highest consumption was observed in the SAMP8 group, with C57BL/6J and SAMR1 exhibiting similar profiles (Figure 2b,h). The lower bone density of C57BL/6J mice at 4 months was strain‐specific: males differed only from SAMP8, while females differed only from SAMR1. It was not until 10 months of age that C57BL/6J females displayed significantly lower bone density compared to both other strains (Figure 2d,j). Notably, all male animals across the strains showed a significant reduction in bone density at 10 months of age compared to their 4‐month‐old counterparts (Figure 2d). However, this pattern was not observed in females; instead, SAMP8 animals demonstrated an increase in bone density over the same period (Figure 2j).

Regarding the peripheral blood glucose levels over time, C57BL/6J males and females exhibited higher glucose levels (Figure 2e,k). SAMP8 animals also displayed higher average glucose levels compared to SAMR1 (Figure 2e). In terms of longevity, SAMP8 males had a shorter lifespan, with a survival proportion of ~60% at 13 months compared to that of the other strains (Figure 2f). Among females, the SAMR1 strain stood out for having shorter longevity, with a survival rate of ~66% compared to 78% for SAMP8 and 100% for C57BL/6J (Figure 2l).

### 3.3. C57BL/6J and SAMP8 Male Animals Show Alterations in Glucose Metabolism

**Figure 3:**
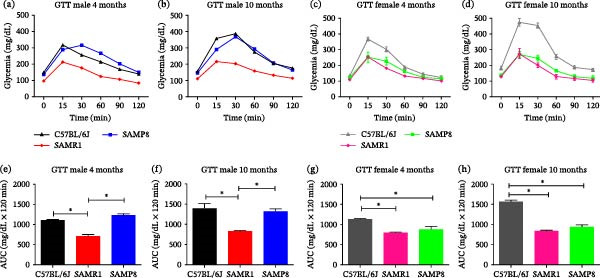
Evaluation of glucose tolerance in C57BL/6J, SAMR1, and SAMP8 mice at 4 and 10 months. (a) GTT for 4‐month‐old males, (b) GTT for 10‐month‐old males, (c) GTT for 4‐month‐old females, (d) GTT for 10‐month‐old females, (e) GTT AUC for 4‐month‐old males, (f) GTT AUC for 10‐month‐old males, (g) GTT AUC for 4‐month‐old females, and (h) GTT AUC for 10‐month‐old females. Results are expressed as the mean ± standard deviation evaluated by one‐way ANOVA Tukey’s test.  ^∗^Represents the statistical difference between the groups, considering *p* < 0.05 (5 animals per group).

**Figure 4:**
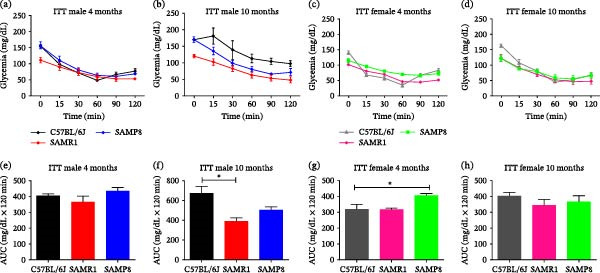
Evaluation of insulin tolerance in C57BL/6J, SAMR1, and SAMP8 mice at 4 and 10 months. (a) ITT for 4‐month‐old males, (b) ITT for 10‐month‐old males, (c) ITT for 4‐month‐old females, (d) ITT for 10‐month‐old females, (e) ITT AUC for 4‐month‐old males, (f) ITT AUC for 10‐month‐old males, (g) ITT AUC for 4‐month‐old females, and (h) ITT AUC for 10‐month‐old females. Results are expressed as the mean ± median and standard deviation evaluated by one‐way ANOVA Tukey’s test.  ^∗^Represents the statistical difference between the groups, considering *p* < 0.05 (5 animals per group).

In vivo glucose metabolism was assessed by GTT and ITT at 4 and 10 months of age to evaluate strain‐specific profiles and aging‐related alterations. Under a 1 g/kg glucose challenge, SAMR1 males exhibited better glucose regulation than the other two strains at both time points (Figure 3a,b,e,f). In contrast, significant differences in glucose handling among females were observed only for C57BL/6J, which differed from SAMR1 at 4 months and from both strains at 10 months (Figure 3c,d,g,h). Following stimulation with 0.75 UI/kg insulin, most parameters were comparable across strains and within each sex at 4 months (Figure 4a,c,e) and 10 months (Figure 4b,d,h). The only differences observed were that C57BL/6J males showed higher ITT responses than SAMR1 at 10 months (Figure 4f), whereas C57BL/6J females exhibited lower ITT responses than SAMP8 at 4 months (Figure 4g).

### 3.4. C57BL/6J, SAMR1, and SAMP8 Show Age‐ and Sex‐Dependent Hematometric Alterations

**Figure 5:**
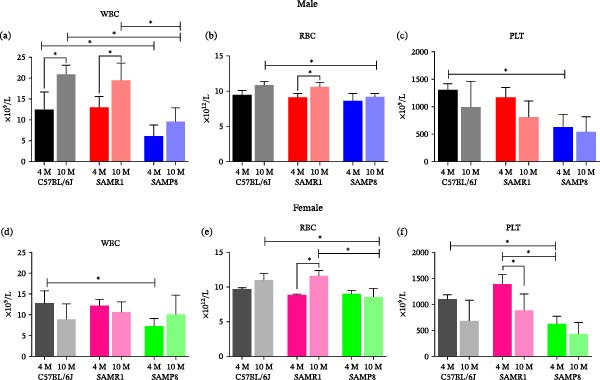
Hematological analysis in C57BL/6J, SAMR1, and SAMP8 mice at 4 and 10 months. (a) WBC count in males, (b) RBC count in males, (c) PLT count in males, (d) WBC count in females, (e) RBC count in females, and (f) PLT count in females. Results are expressed as the mean ± standard deviation evaluated by two‐way ANOVA Tukey’s test.  ^∗^Represents the statistical difference between the groups, considering *p* < 0.05 (5–6 animals per group).

Blood cell counts were performed to physiologically characterize peripheral blood cell populations and their responses to aging. In males, leukocyte count increase between 4 and 10 months in the C57BL/6J and SAMR1 strains relative to their earlier levels, with a higher number of these cells also present in these strains compared to the SAMP8 strain (Figure 5a). In contrast, females showed changes only at the 4^th^ month of life, with C57BL/6J displaying slightly higher leukocyte counts compared to those of the SAMP8 strain (Figure 5d).

Erythrocyte counts were significantly higher at 10 months of age in SAMR1 males and females compared to their earlier values (Figure 5b,e). C57BL/6J (both males and females) and SAMR1 (only females) exhibited higher erythrocyte counts than their respective SAMR1 counterparts (Figure 5b,e).

Platelet counts were higher in both male and female C57BL/6J mice compared to SAMP8 at 4 months of age (Figure 5c,f). In addition, SAMR1 females also showed higher platelet counts than those of SAMP8 females at this age (Figure 5f). By 10 months, platelet levels declined across all strains from their initial values, and no significant differences were observed among the groups (Figure 5f).

**Figure 6:**
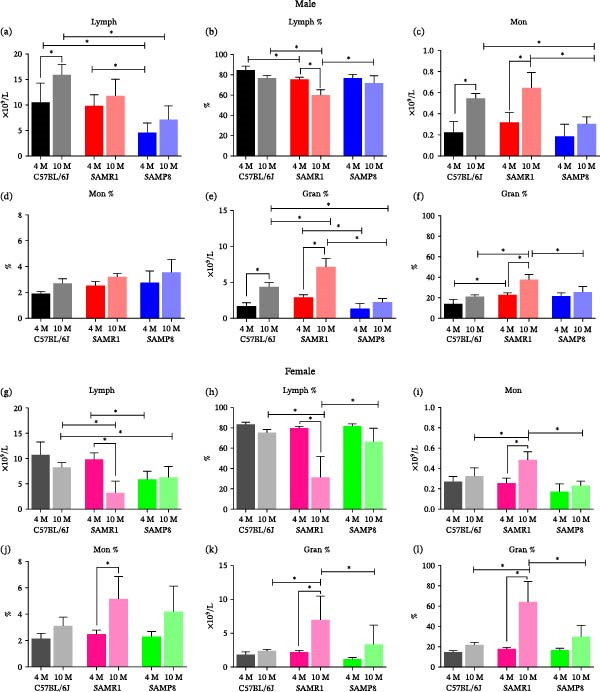
Leukogram analysis in C57BL/6J, SAMR1, and SAMP8 mice at 4 and 10 months. (a) Lymphocyte count (Lymp) in males, (b) Lymphocyte percentage (Lymp%) in males, (c) Monocyte count (Mon) in males, (d) Monocyte percentage (Mon%) in males, (e) Granulocyte count (Gran) in males, (f) Granulocyte percentage (Gran%) in males, (g) Lymp in females, (h) Lymp% in females, (i) Mon in females, (j) Mon% in females, (k) Gran in females, and (l) Gran% in females. Results are expressed as the mean ± standard deviation evaluated by two‐way ANOVA Tukey’s test.  ^∗^Represents the statistical difference between the groups, considering *p* < 0.05 (5–6 animals per group).

Analysis of peripheral immune cell subpopulations revealed that the total number of Lymphs was lower in the SAMP8 strain than in the other two strains. Additionally, lymphocyte numbers increased in C57BL/6J males at 10 months compared to 4 months within the same strain, while SAMR1 females exhibited a reduction over the same period (Figure 6a,g). In terms of the lymphocyte percentage, we observed a decrease in SAMR1 males and females at 10 months compared to 4 months, as well as a reduction at 10 months compared to the other strains (Figure 6b,h).

Monocyte counts, on the other hand, increased in both male and female of the SAMR1 strain, as well as in C57BL/6J males at 10 months compared to 4 months in their respective pairs (Figure 6c,i). Additionally, there was a lower count in SAMP8 males and females compared to SAMR1 and also compared to C57BL/6J in males (Figure 6c,i). The percentage of Mon changed only in SAMR1 females when comparing 10 months of life with 4 months of life (Figure 6d,j).

Granulocyte counts were higher in SAMR1 males and females and in C57BL/6J males when comparing 10 months to 4 months (Figure 6e,k). They also showed higher counts compared to those of C57BL/6J and SAMP8 males and females at 10 months (Figure 6e,k). The same pattern was observed in the percentage of Grans (Figure 6f,l).

**Figure 7:**
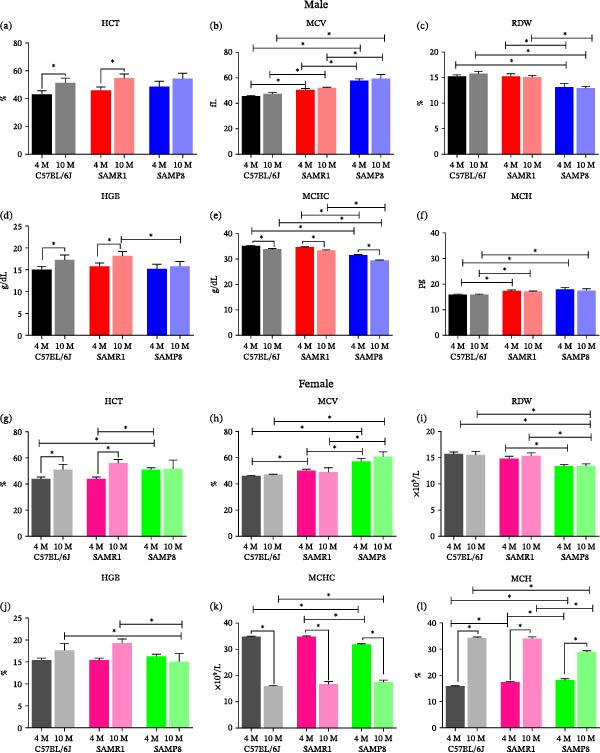
Erythrogram analysis in C57BL/6J, SAMR1, and SAMP8 mice at 4 and 10 months. (a) Hematocrit (HCT) in males, (b) Mean corpuscular volume (MCV) in males, (c) Red cell distribution width (RDW) in males, (d) Hemoglobin (HGB) concentration in males, (e) Mean corpuscular hemoglobin concentration (MCHC) in males, (f) Mean corpuscular hemoglobin (MCH) in males, (g) HCT in females, (h) MCV in females, (i) RDW in females, (j) HGH concentration in females, (k) MCHC in females, and (l) MCH in females. Results are expressed as the mean ± standard deviation evaluated by two‐way ANOVA Tukey’s test.  ^∗^Represents the statistical difference between the groups, considering *p* < 0.05 (5–6 animals per group).

The HCT levels in both male and female C57BL/6J and SAMR1 mice increased significantly at 10 months of age compared to 4 months (Figure 7a,g). Female SAMP8 mice showed a higher HCT percentage at 4 months compared to the other strains (Figure 7g). MCV was reduced in both male and female C57BL/6J mice at both 4 and 10 months when compared to that in the other strains (Figure 7b,h). RDW was slightly lower in both male and female SAMP8 mice compared to that in the other two strains (Figure 7c,i). HGB concentration at 10 months was reduced in SAMP8 compared to SAMR1 male mice, with a slight increase observed in male C57BL/6J and SAMR1 mice at 10 months compared to 4 months (Figure 7d). In females, SAMP8 also showed lower concentrations at 10 months compared to those of C57BL/6J and SAMR1 (Figure 7j). MCHC showed a reduction at 10 months compared to 4 months across all strains in both sexes, with SAMP8 mice exhibiting a greater reduction compared to the other strains at both time points (Figure 7e,k). MCHC values were lower in both male and female C57BL/6J mice compared to the other strains at both 4 and 10 months (Figure 7f,l), with an observed increase in all females at 10 months compared to 4 months within their respective strains (Figure 7f,l).

**Figure 8:**
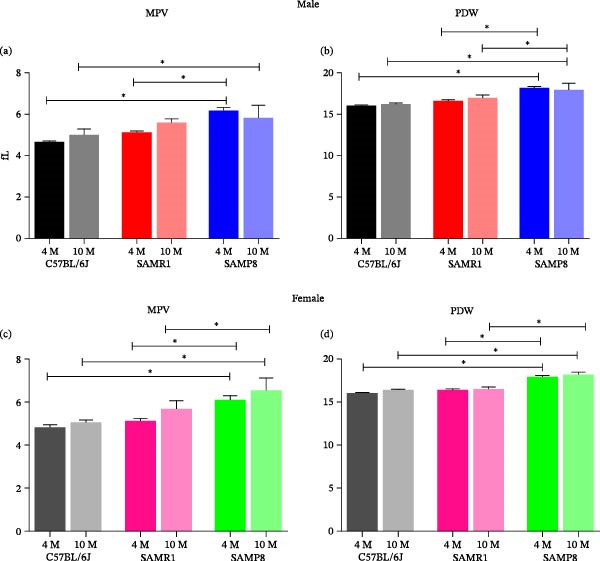
Plateletogram analysis in C57BL/6J, SAMR1, and SAMP8 mice at 4 and 10 months. (a) Mean platelet volume (MPV) in males, (b) Platelet distribution width (PDW) in males, (c) MPV in females, and (d) PDW in females. Results are expressed as the mean ± standard deviation in a two‐way ANOVA Tukey’s test.  ^∗^Represents the statistical difference between the groups, considering *p* < 0.05 (5–6 animals per group).

Finally, analyses of PLTs revealed that SAMP8 males and females exhibit a higher mean platelet volume (MPV) and platelet distribution width (PDW) compared to the SAMR1 and C57BL/6J strains at 4 months of age, with this profile persisting in SAMP8 females at 10 months. However, only C57BL/6J males maintained this profile at 10 months (Figure 8a–dd).

## 4. Discussion

Murine models of aging are divided into two categories: normal aging, with C57BL/6J being the preferred strain, and accelerated aging, with SAMP strains (and SAMR as a control), established by Takeda and collaborators in the 1960s, being among the most important and widely used [1, 8, 9, 17, 18]. For this reason, our study investigated the physiological and metabolic parallels between these strains over 1 year to provide comparative data between these two models used in aging research.

In our analyses, we observed no differences in reproductive parameters among C57BL/6J, SAMR1, and SAMP8 mice, even with the accelerated aging of the latter. This contradicts prior studies suggesting that hormonal changes at 7 months of age should lead to reduced fertility in SAMP8 mice [19–21]. However, our results align with those of Smith et al. [22], who conducted a study on the fertility of SAMP8 male animals, investigating the effects of oxidative DNA damage in sperm. The authors conclude that, despite the high levels of stress and oxidative damage in these cells, fertility was not compromised [22]. Similarly, Tanaka et al. [23] observed no significant differences in the reproduction of SAMP8 animals compared to other strains, such as SAMR1, SAMP6, DDD/Jah, and C57BL/6J, except for a higher number of offspring born per female in the SAMR1 strain, similar to our results. This could be attributed to the larger body size of these animals, potentially allowing for more offspring in the womb during pregnancy [11]. However, we know that several environmental factors, such as housing conditions and nutrition, affect these parameters. Under our experimental conditions, the accelerated senescence of SAMP8 does not seem to be a decisive factor in considering it a model of reproductive aging.

Despite differences in the body size among the strains, with SAMR1 mice being larger and consequently heavier than the other strains, C57BL/6J males consumed less food relative to their body weight. Additionally, both male and female C57BL/6J mice showed lower food intake than that of SAMP8 mice. This pattern was reflected in body weight as SAMR1 males and females were consistently heavier than the other groups, while no significant differences were observed between the C57BL/6J and SAMP8 strains. Growth trajectories were initially similar but began to diverge from the third week of life onward in males and from the sixth week in females, with these differences persisting throughout the experiment, particularly in SAMR1 animals. Similar findings were reported by Pačesová et al. [24] when comparing SAMR1 and SAMP8 strains.

In parallel with the changes in body composition, the skeletal framework undergoes critical age‐related transitions, primarily defined by a reduction in bone mass and structural integrity [25]. With advancing age, bone mass is lost, compromising structural integrity and increasing the risk of osteoporosis. Syed and Melim [26] documented progressive bone mass decline in C57BL/6J beginning at 6 months of age, while Jilka [25] reported a decline in bone quality in SAMP6 animals relative to SAMR1. In addition, Zhang et al. [27] validated SAMP8 as a model of sarcopenia characterized by both muscle mass loss and osteoporosis around 8 months of age, whereas SAMR1 develops sarcopenia without associated bone loss. Our data align with these findings: all male groups exhibited bone loss between months 4 and 10, with C57BL/6J males showing slightly lower bone density than SAMP8. In contrast, females did not exhibit significant age‐related bone loss; instead, SAMP8 females experienced an increase in bone density over time. This sex‐specific pattern likely reflects differences in the hormonal milieu, particularly estrogen, which suppresses bone resorption by inducing osteoclast apoptosis and promotes bone formation by increasing osteoblast survival [28].

Aging is closely associated with changes in the whole‐body energy balance, with glucose metabolism being one of its central components. Glucose tolerance declines with age, as reflected by the high prevalence of type 2 diabetes in the elderly population [29]. This age‐related impairment is linked to both reduced insulin secretion and decreased sensitivity of peripheral tissues to insulin, particularly skeletal muscle, which accounts for ~70% of insulin‐mediated glucose uptake [30, 31]. Therefore, insulin resistance or glucose intolerance is generally accompanied by elevated blood glucose levels, which, in diabetic mice, typically exceed 300 mg/dL [13]. However, this was not observed in any of our groups, although C57BL/6J animals showed higher peripheral blood glucose levels than the other two strains in both males and females. One possible explanation is the mutation in the nicotinamide nucleotide transhydrogenase enzyme found in this strain, a mitochondrial protein strongly associated with glucose metabolism, insulin secretion, and overall mitochondrial function [32–36]. This mutation may also explain why these animals showed impaired glucose handling after glucose administration and a reduced hypoglycemic response after insulin administration in vivo. Notably, the metabolic dysfunction in SAMP8 males coincided with the impaired GTT profiles of C57BL/6J animals at both evaluated intervals.

SAMP8 males responded to the GTT similarly to C57BL/6J males at both time points but showed impaired insulin sensitivity at 10 months. In contrast, female responses differed, with only C57BL/6J females displaying defective responses. These observations support two points. First, metabolic alterations in SAMP8 males may be related to sarcopenia and accelerated senescence, which can disrupt glucose uptake and insulin signaling. Consistent with this, Pačesová et al. [24] reported peripheral insulin resistance in SAMP8 mice at 3 and 6 months, and our own FDG‐PET observations in SAMP8 males [37] corroborate altered glucose uptake. Second, our data reveal pronounced sexual dimorphism across multiple phenotypes, including glucose and insulin metabolism, bone density, survival, and hematological parameters, underscoring that sex is a critical biological variable in aging studies.

The lifespan of animals differs according to genotype and sex, with some strains exhibiting a longer lifespan for females than males, while in others, the opposite occurs, a phenomenon that remains largely unexplained [12]. In this respect, it is curious to note that while C57BL/6J animals showed no difference in survival rates throughout the evaluated period, SAMP8 and SAMR1 animals did. Specifically, SAMP8 males had a nearly 20% higher mortality rate compared to the other two strains, while SAMR1 females had a mortality rate similar to that of SAMP8, contrasting with C57BL/6J, which recorded no deaths during the study period. Some studies, such as that by Musazzi et al. [38], show that there are no significant survival differences between SAMP8 males and females; rather, a distinction exists between the SAMP8 and SAMR1 strains, with the former averaging a lifespan of 9.5–12 months and the latter ranging from 16.5 to 19.5 months. SAMP8 mice show a survival rate of 90%–100% in the first 6 months, which declines to 70%–90% by the ninth month [38]. In contrast, studies by Tanaka et al. [23] show a slight difference in lifespan between SAMR1 and SAMP8 males and females, noting that females tend to live for slightly fewer days than males. In our cohort, SAMR1 females showed a survival pattern similar to that of SAMP8 mice, both of which were inferior to the C57BL/6J group. Environmental factors may have influenced this outcome, and we emphasize that these data should be interpreted with caution given the expected higher mortality typically associated with the SAMP8 strain [19]. Conversely, Ogiso et al. [39] study has observed longer male survival in C57BL/6J relative to females, reinforcing that lifespan depends heavily on experimental facility conditions. Highlighting these interstrain and sex differences in life expectancy is crucial given their implications for aging research design and interpretation.

Because peripheral blood variables are closely interconnected with the progression of aging, monitoring hematological shifts is vital to distinguishing between normal and accelerated senescence [40, 41]. Bayliak et al. [42] evaluated peripheral blood parameters across the lifespan of C57BL/6J mice under every‐other‐day fasting versus ad libitum feeding and reported age‐related decreases in Lymphs and eosinophils alongside increases in neutrophils. In our comparative analysis, male SAMP8 mice exhibited lower total leukocyte, lymphocyte, monocyte, and granulocyte counts compared with C57BL/6J and SAMR1 males, whereas C57BL/6J and SAMR1 males showed increases in these populations from 4 to 10 months. This suggests that peripheral immune profiles of C57BL/6J and SAMR1 males more closely resemble one another than either SAMP8. In a previous study examining lymphoid organs after rapamycin treatment, we observed a classical immunosenescence profile in SAMP8 as early as 5 months, supporting SAMP8 as a model of early immune aging [10]. Together with the comparative C57BL/6J data presented here, these results indicate that SAMP8 is an appropriate model for immunosenescence studies, with SAMR1 functioning as a useful control.

The idea that senescence might lead to anemia, either through iron deficiency or a reduction in the production of new RBC by the bone marrow, prompted Magnani et al. [43] to conduct a study with BALB/c mice in the 1980s. Their results indicated that anemia was not detected in senescent mice, suggesting that, even in elderly animals, erythropoiesis activity remained efficient [43]. Any alterations associated with the aging process appeared to be compensated for by the organism, stabilizing the red blood cell parameters of older animals to levels comparable to those of young ones [43]. Similar findings were reported in young (8–12 weeks) and elderly (30–35 weeks) SAMP1 animals investigated for erythropoiesis, with no apparent differences between the age groups [44]. Conversely, Kong et al. [45] conducted a comparative study of hematimetric parameters among three different strains, C57BL/6J, BALB/c, and ICR, and observed that C57BL/6J animals had a tendency for higher erythrocyte and leukocyte counts. This may be attributed to their immune system being more predisposed to a Th1 profile in both genders [45]. In contrast, our results reveal that C57BL/6J and SAMR1 animals share very similar profiles in the leukocyte series and platelet parameters, which differ from SAMP8, with some differences noted between genders. Red blood cell parameters were less stable, with alterations in all groups across both evaluated periods for both males and females, with females showing more pronounced changes. We hypothesize that, unlike the leukocyte series, which is vital for the organism’s defense, the red blood cell series does not require such fine‐tuning, as long as it remains functional to fulfill its role. This makes it more susceptible and adaptable to variations in its parameters, which, as noted by Etim et al. [40], can be affected by various factors such as age, nutrition, animal health, physical activity level, sex, and environmental factors.

Both male and female SAMP8 mice exhibited increased MPV compared to C57BL/6J and SAMR1. Elevated MPV may serve as an indirect marker of low‐grade chronic inflammation, a hallmark of aging demonstrated in SAMP models, because larger PLTs are metabolically and enzymatically more active, contain more dense granules, and express higher levels of adhesion molecules (e.g., P‐selectin), thereby promoting the release of proinflammatory cytokines. Thus, elevated MPV often correlates with increased circulating inflammatory cytokines [46]. Supporting this interpretation, previous data showed no difference in MPV between SAMP8 and SAMR1 at 5 months [10], whereas in our analyses, this difference is evident at 4 and 10 months; in contrast, C57BL/6J diverges earlier, from 4 months onward. Together, these findings are consistent with an accelerated, inflammation‑associated aging trajectory in SAMP8 relative to strains that display the more typical, gradual age‑related changes.

Since our study was primarily designed to capture systemic and phenotypic changes over time, it does not focus on the underlying cellular and molecular mechanisms driving the observed differences. Future investigations, incorporating more in‐depth immunophenotyping, bone marrow, and hematopoietic analyses, as well as hormonal, inflammatory, and omics‐based approaches, will be valuable to further elucidate these mechanisms. Such studies will not only expand the framework established here but also help validate and refine the biological interpretations of the phenotypic patterns we report, strengthening the use of these models in aging research.

## 5. Conclusion

In summary, our data indicate that C57BL/6J and SAMR1 share more similar biological aging phenotypes than either does with SAMP8. The SAMP8 strain shows features consistent with accelerated aging, including altered immune parameters, increased MPV, and sex‐specific metabolic impairments, whereas SAMR1 behaves more similarly to the naturally aged C57BL/6J strain under our experimental conditions. These strain‐ and sex‐dependent differences have important implications for model selection in aging research and underscore the need for careful control selection and parallel testing with naturally aging wild‐type strains when employing accelerated‐aging models.

## Author Contributions

Luiz Adriano Damasceno Queiroz, Renata Spalutto Fontes, Stephen Fernandes Rodrigues, and Joilson O. Martins conceived and designed the experiments. Luiz Adriano Damasceno Queiroz, Renata Spalutto Fontes, Mariana de Araujo Oliveira, Kamilla Costa Pantoja, Josiane Betim Assis, Ywa Perpetuo Socorro Toda Tavares, Rafael dos Santos Barros, Walter Miguel Turato, and Anderson Sá‐Nunes performed the experiments. Luiz Adriano Damasceno Queiroz, Anderson Sá‐Nunes, Naima Moustaid‐Moussa, Stephen Fernandes Rodrigues, and Joilson O. Martins analyzed the data. Stephen Fernandes Rodrigues, Anderson Sá‐Nunes, and Joilson O. Martins contributed with reagents/materials/analysis tools. Luiz Adriano Damasceno Queiroz and Joilson O. Martins wrote the paper with the assistance of all the authors.

## Funding

The authors are supported by the Fundação de Amparo à Pesquisa do Estado de São Paulo (FAPESP) (Grants 2020/05439‐4, 2022/00482‐4, 2022/02742‐3, 2023/05115‐2, and 2024/00810‐7), the Conselho Nacional de Desenvolvimento Científico e Tecnológico (CNPq) (Grants 310993/2020‐2, 312674/2021‐0, and 301423/2025‐3), and the Coordenação de Aperfeiçoamento de Pessoal de Nível Superior (CAPES) (Finance Code 001).

## Conflicts of Interest

The authors declare no conflicts of interest.

## Data Availability

The data in this study are available upon reasonable request to the corresponding authors (Dr. Stephen F. Rodrigues, stephen.rodrigues@usp.br and/or Joilson O. Martins, martinsj@usp.br).
